# Dissemination of Carbapenemases (OXA-48, NDM and VIM) Producing *Enterobacteriaceae* Isolated from the Mohamed VI University Hospital in Marrakech, Morocco

**DOI:** 10.3390/antibiotics10050492

**Published:** 2021-04-26

**Authors:** Souad Loqman, Nabila Soraa, Seydina M. Diene, Jean-Marc Rolain

**Affiliations:** 1 Laboratoire de Lutte Contre les Maladies Infectieuses, Faculté de Médecine et de Pharmacie, Université Cadi Ayyad, Marrakech 40000, Morocco; na.soraa@uca.ma; 2 Laboratoire de Microbiologie, CHU Mohammed VI, Av Ibn Sina Amerchich, BP 2360, Marrakech 40000, Morocco; 3 Aix Marseille Université, IRD, APHM, MEPHI, IHU-Mediterranée Infection, 13385 Marseille, France; seydina.diene@univ-amu.fr (S.M.D.); jean-marc.rolain@univ-amu.fr (J.-M.R.)

**Keywords:** *Enterobacteriaceae*, carbapenem resistance, OXA-48, NDM-1, VIM

## Abstract

The emergence and spread of carbapenem-resistant *Enterobacteriaceae* (CRE) represent a major clinical problem and raise serious health concerns. The present study aimed to investigate and ascertain the occurrence of CRE among hospitalized patients of Mohamed VI University Hospital, Marrakech, Morocco. Biological samples were collected over a one-year period (2018). The bacterial isolates were identified by MALDI-TOF-MS. Antibiotic susceptibility testing was performed using disc diffusion and Etest. The modified Hodge test and combined disc diffusion test were used for phenotypic detection. CRE hydrolyzing enzyme encoding genes: *bla*OXA-48, *bla*KPC, *bla*IMP, *bla*VIM, and *bla*NDM were characterized by PCR and DNA sequencing. In total, 131 non-duplicate CRE clinical strains resistant to Ertapenem were isolated out of 1603 initial *Enterobacteriaceae*. *Klebsiella pneumoniae* was the most common species (59%), followed by *Enterobacter cloacae* (24%), *E. coli* (10%), *Citrobacter freundii* (3%), *Klebsiella* *oxycota* (2%), *Serratia marcescens* (1%), and *Citrobacter braakii* (1%). Of these, 56.49%, 21.37%, 15.27%, 3.38%, and 3.05% were collected from blood, urine, pus, catheters and respiratory samples, respectively. Approximately 85.5% (112/131) of the isolates were carbapenemase producers (40 *bla*OXA-48, 27 *bla*NDM, 38 *bla*OXA-48 + *bla*NDM and 7 *bla*VIM). All metallo-β-lactamases isolates were NDM-1 and VIM-1 producers. This is the first documentation of *bla*OXA-48 genes from *C. freundii* and *C. braakii* in Morocco.

## 1. Introduction

Over the past few years, carbapenem-resistant *Enterobacteriaceae* (CRE), the most frequent pathogens responsible for nosocomial infections, have emerged and rapidly spread all over the world, posing great challenges to human health [1,2,3,4,5,6,7]. Conventionally, carbapenem antibiotics have been considered the most effective among available antibacterial agents for the treatment of infections caused by *Enterobacteriaceae* (e.g., *E. coli* and *Klebsiella pneumoniae*), and are still being used as the antibiotics of last-resort to date [8,9]. Recently, these last-line therapeutic agents have lost effectiveness in more than half of the people treated, across many countries, leading to treatment failure and reducing clinical treatment options [10,11]. According to the latest data reported by the Chinese Antimicrobial surveillance network (www.chinets.com, accessed on 29 September 2018), the resistance rates of *K*. *pneumoniae* to meropenem and imipenem (two parenteral carbapenems) were eight times higher in 2018 compared to 2005 (2.9%–26% and 3%–25%, respectively). Moreover, carbapenem-resistant *K. pneumoniae* has been recently recognized as life-threatening in Europe and is even listed on the global priority list, established by the World Health Organization, of antibiotic-resistant bacteria for which new antibiotics are urgently needed [12,13].

There are three major mechanisms responsible for carbapenem resistance in *Enterobacteriaceae*: enzyme production (cephalosporinases, extended-spectrum β-lactamases (ESBLs), metallo-β-lactamases (MBLs) and carbapenemases), efflux pumps and porin mutations [14,15].

The production of carbapenemases, a type of β-lactamase hydrolyzing enzymes, is considered a major mechanism conferring carbapenem resistance for CRE and contributes largely to the development and spread of antibiotic resistance [16]. Carbapenemase encoding genes, often located in plasmids, are associated with mobile elements that facilitate their acquisition (e.g., by clonal and horizontal transfer) and spread from bacteria to bacteria [17]. KPC, VIM, IPM, NDM and OXA-48 are examples of the most common resistant genes widely distributed among *Enterobacteriaceae* clinical isolates. The *bla*OXA-48, first identified from carbapenem-resistant *K. pneumoniae* isolates in Istanbul, Turkey, has since been extensively reported as a source of nosocomial infection outbreaks in many parts of the world, notably from countries surrounding the Mediterranean area [18,19,20]. North African countries (mainly Egypt, Libya, Tunisia and Morocco) are considered the principal reservoirs of OXA-48 producing *Enterobacteriaceae* [21,22,23,24]. In Morocco, the situation of carbapenem-resistant *Enterobacteriaceae* has been the subject of only a few and outdated studies [25,26]. Most current and available research is confined to case reports or research studies that have focused on few patient numbers, for specific hospital units, specific bacterial species and specific genes, over a short period of time [27,28,29,30]. Thus, we conducted this one-year surveillance study (2018) to characterize the dissemination and characteristics of carbapenem-resistant *Enterobacteriaceae* (including *bla*OXA-48, *bla*KPC, *bla*IMP, *bla*VIM, and *bla*NDM) among hospitalized patients, from different units, in Mohamed VI University Hospital, Marrakech, Morocco.

## 2. Results

A total of 131 non-duplicate CRE resistant to different carbapenems were isolated and identified out of 1603 initial *Enterobacteriaceae* clinical strains. The majority were isolated from patients in the neonatal unit (40.3%), followed by the plastic surgical unit (14.1%), urology-nephrology unit (11.1%), pediatric reanimation unit (13%), medical-surgical resuscitations (11.1%) and finally, the medicine unit (10.1%). The age of these patients ranged from 0 months to 84 years. Of these, 57% were children (0–15 years) and 43% adults (≥16–80 years), with 58 (44.27%) females and 73 (55.73%) males in total.

**Figure 1:**
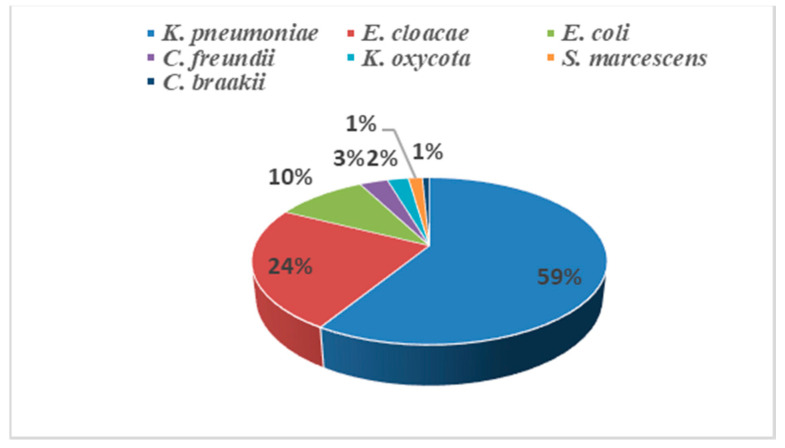
Distribution of the 131 isolated CRE.

*Klebsiella pneumoniae* was the most common isolated species (59%, 77/131) (Figure 1), followed by *Enterobacter cloacae* (24%, 31/131), *E. coli* (10%, 13/131), *Citrobacter freundii* (3%, 4/131), *Klebsiella oxycota* (2%, 3/131), *Serratia marcescens* (1%, 2/131) and *Citrobacter braakii* (1%, 1/131). The strains were isolated from various clinical specimens, including blood (56.49%, 74/131), urine (21.37%, 28/131), pus (15.27%, 20/131), catheters (3.38%, 5/131) and respiratory samples (3.05%, 4/131).

Most of the CRE strains (>83%) were resistant to ertapenem, imipenem, gentamicin, ciprofloxacin, trimethoprim-sulfamethoxazole, and piperacillin-tazobactam (Table 1). When all 131 CRE isolates were considered; 100% were resistant to ETP, GEN and PTZ, 87% were resistant to SXT, 86.4%were resistant to CIP and 82.6% were resistant to IMP. Only 34% of the strains were resistant to colistin. All the strains were resistant to three functional drug classes simultaneously, 100% resistant to ETP, GEN and PTZ, which means they are multidrug-resistant. Approximately 98.7% of *K. pneumoniae* isolates were multidrug-resistant, while 21 of them were resistant to all tested antibiotics. All isolated strains of *E. cloacae* and *E. coli* were found to be multidrug-resistant. Fifteen of the 31 *E. cloacae* and five out of the 13 *E. coli* were resistant to all tested antibiotics.

Approximately 85.5% (112/131) of the isolated strains were carbapenemase producers (Table 2). Of these, 40 were positive for *bla*OXA-48, 27 for *bla*NDM and 7 for *bla*VIM, as determined by PCR. In 38 strains, both *bla*OXA-48 and *bla*NDM carbapenemase genes were identified. All metallo-β-lactamases isolates were NDM-1 and VIM-1 producers. IMP and KPC genes were not detected in any of our isolates. *K. pneumoniae* was the most common carbapenemase gene producer (61.6%, 69/112), followed by *E. cloacae* (21.42%, 24/112) and *E. coli* (9.82%, 11/112). Over 33.7% (26/77) of the *K. pneumoniae* isolates possessed *bla*OXA-48 and *bla*NDM simultaneously. While in only 19.35% (6/31) of *E. cloacae* and 23.07% (3/13) of *E. coli* were the two genes detected alongside each other.

Phenotypic detection by MHT correctly identified carbapenemase producers with a sensitivity of 96% and specificity of 100%.

## 3. Discussion

The emergence and dissemination of carbapenem-resistant *Enterobacteriaceae* represent a major clinical problem often associated with significant morbidity and mortality rates, due to treatment failure and the limitation of alternative therapeutic options. Early monitoring of CRE emergence and characterization of the types of carbapenemases produced is of great value for preventing and controlling the spread of CRE in hospital settings.

In the present study, a total of 131 non-duplicate CREs resistant to different carbapenems were isolated and identified out of 1603 initial *Enterobacteriaceae* clinical strains (8.17%). This prevalence is three times higher than that recorded in a similar study, conducted by Wartiti et al. in Rabat, Morocco [31]. In the United States, the prevalence of CRE is generally around 1.4–4.2% [32,33]. In a study in the Asia–Pacific region, CRE isolates rate causing intra-abdominal infections was two times higher (16.5%) compared to our study [34]. On a regional scale, more specifically, in the Mediterranean area, the prevalence of carbapenemases is usually high and variant among countries, partially depending on the population exchange relationship between the regions and the possible reservoirs of each carbapenemase [19].

Indeed, the acquirement of CRE infections is highly related to certain risk factors, such as: being transferred between hospitals or from long-term care centers; a long stay in the hospital, especially in an intensive care unit; having a surgical operation; transplantation; antibiotics usage; central venous catheter presence; having diabetes mellitus [35,36]. In this study, the majority of CRE isolates were from patients in the in the neonatal unit (40.3%), followed by the plastic surgical unit (14.1%), urology-gy-nephrology unit (11.1%), pediatric reanimation unit (13%), medical-surgical resuscitations (11.1%) and finally the medicine unit (10.1%).

Among the isolated strains, *K. pneumoniae* was the most common species isolated in our study (59%), followed by *E. cloacae* (24%), then *E. coli* (10%). This distribution of CRE species is consistent with that of other CRE studies in the United States and Europe [32,37]. Additionally, in a recent study conducted in Tunisian and Libyan hospitals, *K. pneumoniae* and *E. coli* were the most isolated strains [23]. This is in concordance with the CDC’s recommendations that *K. pneumoniae*, *E. coli* and *Enterobacter* spp. are the key healthcare-associated pathogens to focus on in the control of the CRE epidemic [38]. In the Asia–Pacific region, a contrary trend was observed in the study conducted by Jean et al. [34], where *Enterobacter* species, *K. pneumoniae* and *E. coli* were the three major pathogens isolated with 77.4%, 40.9% and 11.7%, respectively. At the national level, the study of Wartiti et al. [31] revealed that *E. coli* isolates accounted for 63.9%, followed by *Klebsiella* spp. Accounting for 27.9% and *Enterobacter* spp. for 8.2%.

The majority of CRE isolates in our study were collected from the blood (56.49%), urine (21.37%), pus (15.27%), catheters (3.82%) and respiratory samples (3.05%). In comparison to other studies, urine and blood samples are usually the most common sources of CRE isolates, while other sources such as wound discharge and sputum were the least [1,39].

More than 83% of the CRE isolates were resistant to different classes of antibiotics tested, typically ertapenem, imipenem, gentamicin, ciprofloxacin, trimethoprim-sulfamethoxazole and piperacillin-tazobactam. A similar antibiotic susceptibility profile was reported by Wartiti et al. [31], except for imipenem found non-susceptible in our case. In contrast, Mathlouthi et al. [23] observed a low resistance for imipenem in Tunisian and Libyan hospitals.

Carbapenemases were detected in 85.5% (112/131) of the CRE isolates (Table 2)**.** Of these, 40 were positive for *bla*OXA-48, 27 for *bla*NDM and 7 for *bla*VIMas, as determined by PCR. In 38 strains, both *bla*OXA-48 and *bla*NDM carbapenemase genes were identified. The 19 remaining isolates (14.50%), negative for carbapenemase genes detection, are likely to not be susceptible to carbapenem due to a combination of cephalosporins with a weak carbapenemase activity gene [40], or simply the disposal of other carbapenemases that were not assessed in this study, such as *bla*GES or *bla*NMC-A, although these have not been reported to date in the country. OXA-48 is the most prevalent carbapenemase gene detected in Morocco, as is the case in the present work. After its initial identification from a *K. pneumoniae* isolates in Rabat in 2010, from a 60-year-old man with no previous medical history [24], OXA-48 producers have intensively become the most CRE responsible for nosocomial infections in our country [28,30,41]. Indeed, North African countries (mainly Egypt, Libya, Tunisia and Morocco) are considered the principal reservoirs of OXA-48 producing *Enterobacteriaceae* [21,22,23,24]. The *bla*OXA-48 gene identified in Morocco from *K. pneumoniae* was located inside Tn1999, a composite transposon made of two copies of the insertion sequence IS1999, which is itself always identified on a ca. 62 kb plasmid that is the main vehicle for *bla*OXA-48 dissemination [41]. It is worth mentioning here, that a closely related or identical OXA-48 gene to the one identified in Morocco was later recognized as a potential source of OXA-48 dissemination in the Netherlands and France [42,43].

Initially, and most frequently, the *bla*OXA-48 genes were identified in *K. pneumoniae*. However, many other CRE species have been recently recognized as OXA-48 producers, such as *Enterobacter* spp., *K. oxytoca*, *E. coli*, *C. freundii*, *S. marcescens*, etc. In the present study, the majority of *bla*OXA-48 genes were produced by *K. Pneumoniae* (25/40), *E. cloacae* (10/40) and *E. coli* (4/40). In Morocco, the blaOXA-48 genes were previously identified in different species, such as *K. pneumoniae*, *K. oxytoca*, *E. coli*, *E. cloacae* and *M. morganii* [24,25,26,28,41]. According to our best knowledge, this is the first detection of *bla*OXA-48 genes from *C. freundii* and *C. braakii* in the country.

*Citrobacte*r spp., mostly *C. freundii*, are known as opportunistic pathogens, accountable for multiple healthcare-associated infections, including respiratory infections and urinary tract infections. In a recent study conducted by Arana et al. [44] in Spain, 57 *C. freundii* and one *C. braakii* out of 119 isolates of *Citrobacter* spp. tested were found to be carbapenemase producers (58.7% VIM-1, 31.7% OXA-48, 19% KPC-2, 3.2% NDM-1 and 1.6% VIM-2). *C. freundii* produced up to five carbapenemase types simultaneously, including VIM-1 and OXA-48 co-production. The study of Arana et al. also highlighted several remarkable findings, such as clonal and polyclonal dissemination of *C. freundii* across different geographical areas and hospitals. OXA-48 and VIM-1 enzymes encoded were also detected in *C. freundii* from patients with acute leukemia in Spain [45].

NDM gene was the second most detected carbapenemase in our study (27/112). This gene (NDM-1, New Delhi metallo-β-lactamase-1), first identified in a *K. pneumoniae* strain from a Swedish patient previously hospitalized in India, has since been extensively reported as a source of nosocomial infection outbreaks in many parts of the world, including Morocco [46]. Most of the NDM-1 producer isolates in the country were in a hospital setting [28]. In our study, the majority of *bla*NDM-1 genes were produced by *K. pneumoniae* (13/27), *E. cloacae* (7/27) and *E. coli* (4/27). Over 26 *K. pneumoniae*, 6 *E. cloacae* and 3 *E. coli* isolates, were found to be positive for both the *bla*NDM-1 and the *bla*OXA-48 genes. As with the *bla*OXA-48 gene, NDM-1 was initially and most frequently identified from *K. pneumoniae*, but has recently been increasingly reported in other species [47]. Being encoded on highly mobile conjugative plasmids, NDM enzymes are easily transferred between bacteria of inter-and intraspecies, especially by horizontal transfer, which facilitates their dissemination [48].

Other carbapenemase genes have been detected in Morocco, such as KPC, IMP, and VIM, but at a low rate compared to OXA-48 and NDM-1 [49,50]. In our study, VIM was identified in only seven isolates (5 *K. pneumoniae*, one *E. cloacae* and one *C. freundii*). No isolate scored positively for KPC and IMP production. In Morocco, IMP carbapenemase producers were essentially isolated from community-acquired infections, while NDM-producers were identified in hospital settings [25,49].

## 4. Material and Methods

### 4.1. Bacterial Strains

From January to December 2018, over 131 non-duplicate Gram-negative clinical isolates with reduced susceptibility to carbapenem antibiotics were collected out of 1603 initial *Enterobacteriaceae* from hospitalized patients in Mohammed VI University Hospital, Marrakech. These CRE strains were isolated from urine, pus, respiratory samples, blood and catheters. Species identification was previously carried out using rapid biochemical and bacterial tests, Phoenix automated microbiology system (Becton–Dickinson Diagnostic Systems, Sparks, MD, USA) and further confirmed by matrix-assisted laser desorption and ionization time-of-flight mass spectrometry (MALDI-TOF-MS) (MicroflexTM; Bruker Daltonic, Bremen, Germany).

### 4.2. Antibiotics Susceptibility Testing

Antibiotic susceptibility was determined on Mueller–Hinton agar using the standard disk diffusion method, as described by the European Committee on Antimicrobial Susceptibility Testing (EUCAST) 2017 and also using the Phoenix system. Seventeen antibiotics (bioMérieux, Marcy L’Etoile, France) were tested, namely, amoxicillin (AMX), amoxicillin/clavulanate (AMC), piperacillin/tazobactam (PTZ), cephalothin (CEF), ceftriaxone (CRO), cefepime (CPM), ertapenem (ETP), imipenem (IMP), amikacin (AMK), gentamicin (GEN), ciprofloxacin (CIP), Fosfomycin (FOS), nitrofurantoin (NIT), doxycycline (DCI), trimethoprim/sulfamethoxazole (SXT) and colistin (CS). Sensitivity to imipenem, ertapenem, meropenem (MEM) and colistin was confirmed by the antimicrobial gradient method Etest (bioMérieux, Marcy L’Etoile, France) in collected isolates.

### 4.3. Phenotypic Detection of Carbapenemases

The detection of carbapenemase production was characterized by two different methods: modified Hodge test (MHT) and combined disc diffusion method (metallo-β-lactamases (MBL), KPC, Ampc and OXA-48). The phenotypic detection of MBL producers by the combined disc diffusion method is based on the specific inhibition of MBLs by chelating agents. Briefly, on Mueller–Hinton agar a carbapenem disc (imipenem 10 µg, Oxoid, Hants, UK) was placed alone and in combination with an MBL inhibitor (EDTA (Sigma-Aldrich, Darmstadt, Germany), 292 µg added), KPC inhibitor (phenylboronic acid, PBA (Sigma-Aldrich), 400 µg added), cloxacillin 250 µg (AmpC inhibitor) and temocillin 30 µg (for OXA-48-like screening). The discs were placed 25 mm apart. Generated inhibition zones around the imipenem-inhibitor discs were compared to that of the imipenem disc alone. Strains showing more than 5 mm zone diameter around the imipenem with EDTA disc and the one with imipenem alone were considered MBL producers. Strains showing more than 5 mm zone diameter around the imipenem disc alone and in combination with cloxacillin were considered positive for AmpC production. If the inhibition zone difference was more than 5 mm with the imipenem plus PBA but there was no difference (<5 mm) with the imipenem plus cloxacillin, then this was an indication of the KPC enzyme. Negative results of all test synergy alongside a zone of inhibition with temocillin (30 µg) were presumptive of an OXA-48-like enzyme.

### 4.4. Molecular Characterization of Carbapenemase Genes

DNA was extracted from the different CRE isolates using the boiling lysis method [51]. Briefly, each strain was streaked and cultured on Luria Bertani agar, before overnight incubation at 37 °C. One colony of bacterial growth was suspended in 200 µL of nuclease-free water (Invitrogen, Paisly, UK). The suspension was boiled at 100 °C for 10 min in a thermal block (Polystat 5, Bioblock Scientific, Illkirch, France), then centrifuged at 19,000× *g* for 5 min. An aliquot of DNA supernatant (5 µL) was used directly as a template for PCR assay.

All CRE isolates were tested for the presence of carbapenemases genes by real-time PCR (BioRad CFX96 Real-Time System, Marnes-la-Coquette, France). Several carbapenemase genes (*bla*NDM, *bla*VIM, *bla*IMP, *bla*KPC, and *bla*OXA-48) were screened with specific primers listed in Table 3.

PCR positive products were purified and sequenced using the Big Dye terminator chemistry on an ABI 3130XL automated sequencer (Thermo Fisher Scientific, Waltham, MA, USA). Obtained sequences were analyzed using Codon Code Aligner software (version 3.7.1.1) and then examined using the BlastN and BlastP compared against the NCBI database (www.ncbi.nlm.nih.gov, accessed on accessed on 29 September 2018) and ARG-ANNOT (Antibiotic Resistance Gene-ANNOTation).

## 5. Conclusions

In conclusion, in the present study, a total of 131 non-duplicate CREs were isolated and characterized from hospitalized patients of Mohamed VI University Hospital, Marrakech, Morocco. *K. pneumoniae*, *E. cloacae*, and *E. coli* were the most common strains among the different isolated CRE. More than 83% of the isolates were resistant to different classes of antibiotics tested, typically gentamicin, piperacillin-tazobactam, and ertapenem. Carbapenemase genes were detected in 85.5% (112/131) of the CRE isolates. Of these, 40 were positive for *bla*OXA-48, 27 for *bla*NDM, and 7 for *bla*VIM, as determined by PCR. In 38 strains, both *bla*OXA-48 and *bla*NDM carbapenemase genes were identified. OXA-48 and NDM were the most prevalent carbapenemase genes among *K. pneumoniae*, *E. cloacae* and *E. coli* isolates. This study reports the occurrence of OXA-48 in *C. freundii* and *C. braakii* for the first time in the country. Moreover, it represents the first documentation of the emergence of *bla*NDM and *bla*VIM genes in Marrakech hospitals. The alarming spread of these isolates will seriously limit options for clinical treatment in the future. Such findings emphasize the urgent need for control precautions to be taken to prevent the further spread of CRE.

## Figures and Tables

**Table 1:** Antimicrobial susceptibility results of the most commonly isolated carbapenem-resistant *Enterobacteriaceae* strains.

Antibiotics *	All (*n* = 131)	*K. pneumoniae* (*n* = 77)	*E. cloacae* (*n* = 31)	*E. coli* (*n* = 13)
ETP	100% (*n* = 131)	98.7% (*n* = 76)	100% (*n* = 31)	100% (*n* = 13)
IMP	83.21% (*n* = 109)	80.5% (*n* = 62)	84% (*n* = 26)	76.92% (*n* = 10)
GEN	100% (*n* = 131)	100% (*n* = 77)	100% (*n* = 31)	100% (*n* = 13)
CIP	87.02%(*n* = 114)	84.42% (*n* = 65)	93.55% (*n* = 29)	84.61% (*n* = 11)
SXT	87.79% (*n* = 115)	88.30 (*n* = 68)	93.55% (*n* = 29)	76.92% (*n* = 10)
PTZ	100% (*n* = 131)	100% (*n* = 77)	100% (*n* = 31)	100% (*n* = 13)
CS	34.35% (*n* = 45)	27.27% (*n* = 21)	48.39% (*n* = 15)	38.46% (*n* = 5)

* ETP (ertapenem); IMP (imipenem); GEN (gentamicin); CIP (ciprofloxacin); SXT (trimethoprim/sulfamethoxazole); PTZ (piperacillin/tazobactam); CS (colistin).

**Table 2:** Prevalence of carbapenemase genes among the isolated strains.

Species	*bla*OXA-48	*bla*NDM	*bla*KPC	*bla*VIM	*bla*OXA-48 *+ bla*NDM	*bla*OXA-48 *+ bla*KPC	*bla*NDM *+ bla*KPC	*bla*NDM *+ bla*VIM	Unknown	Total
*K. pneumoniae* (*n* = 77)	25	13	0	5	26	0	0	0	8	77
*K. oxytoca* (*n* = 3)	1	1	0	0	0	0	0	0	1	3
*E. cloacae* (*n* = 31)	10	7	0	1	6	0	0	0	7	31
*E. coli* (*n* = 13)	4	4	0	0	3	0	0	0	2	13
*C. freundii* (*n* = 4)	0	1	0	1	2	0	0	0	0	4
*C. braakii* (*n* = 1)	0	0	0	0	1	0	0	0	0	1
*S. marcescens* (*n* = 2)	0	1	0	0	0	0	0	0	1	2
Total	40	27	0	7	38	0	0	0	19	131

**Table 3:** List of primers and probes.

Target Gene	Type of PCR	Primer Name	Primers/Sond	Amplicon Size (bp)	Ref.
OXA-48	Standard PCR	OXA48_STD_F	TTGGTGGCATCGATTATCGG	744	[52]
		OXA48_STD_R	GAGCACTTCTTTTGTGATGGC		
	Real-time PCR	OXA48_RT_F	TCTTAAACGGGCGAACCAAG	125	[53]
		OXA48_RT_R	GCGTCTGTCCATCCCACTTA		
		OXA48_RT_Probe	6-FAM-AGCTTGATCGCCCTCGATTTGG-TAMRA		
NDM	Standard PCR	NDM-1-F	CATTTGCGGGGTTTTTAATG	1022	[54]
		NDM-1-R	CTGGGTCGAGGTCAGGATAG		
	Real-time PCR	NDM_RT_F	GCGCAACACAGCCTGACTTT	155	[54]
		NDM_RT_R	CAGCCACCAAAAGCGATGTC		
		NDM_RT_Probe	6-FAM-CAACCGCGCCCAACTTTGGC-TAMRA		
KPC	Standard PCR	KPC-F	ATGTCACTGTATCGCCGTCT	893	[54]
		KPC-R	TTTTCAGAGCCTTACTGCCC		
	Real-time PCR	KPC-F	GATACCACGTTCCGTCTGGA	180	[23]
		KPC-R	GGTCGTGTTTCCCTTTAGCC		
		KPC-Probe	6-FAM-CGCGCGCCGTGACGGAAAGC-TAMRA		
VIM	Standard PCR	VIM-F	TGGTCTACATGACCGCGTCT	766	[55]
		VIM-R	CGACTGAGCGATTTGTGTG		
	Real-time PCR	VIM-F	CACAGYGGCMCTTCTCGCGGAGA	132	[53]
		VIM-R	GCGTACGTYGCCACYCCAGCC		
		VIM-Probe	6-FAM-AGTCTCCACGCACTTTCATGACGACCGCGTCGGCG-TAMRA		
IMP	Standard PCR	IMP-F	CATGGTTTGGTGGTTCTTGT	488	[56]
		IMP-R	ATAATTTGGCGGACTTTGGC		

## Data Availability

Data is contained within the article.
